# Alicaforsen: An Emerging Therapeutic Agent for Ulcerative Colitis and Refractory Pouchitis

**DOI:** 10.14740/gr599w

**Published:** 2014-05-02

**Authors:** Terron Anthony Hosten, Ke Zhao, Hong Qiu Han, Gang Liu, Xiang Hui He

**Affiliations:** a Department of General Surgery, Tianjin Medical University General Hospital, Tianjin, China

**Keywords:** Alicaforsen, Refractory pouchitis, Ulcerative colitis

## Abstract

Pouchitis is a relatively common complication that develops following ileal pouch-anal anastomosis in patients with complicated ulcerative colitis (UC). Both pouchitis and UC share similarities in their development, as well as in the mechanisms involving mediators of the inflammatory process. In the recent years, the discovery and investigation of biological therapies have led to advancement in the management of these disorders, and the continuation of research on this novel area holds strong implications for a future reduction in the use of invasive surgical procedures. Alicaforsen represents one of these emerging therapeutic agents, and has demonstrated promising results in both preclinical and clinical settings. This article reviews the therapeutic effects of alicaforsen for the management of UC and refractory pouchitis, with special emphasis on the mechanism of action of this therapeutic agent and the clinical studies asserting its effectiveness.

## Introduction

Ulcerative colitis (UC) continues to be a major illness affecting the quality of life of many patients worldwide. Not only this condition slowly growing in prevalence, but also the complications of surgical treatment, especially pouchitis, have become a growing issue of concern. Ileal pouch-anal anastomosis (IPAA) following total proctocolectomy (TPC) is considered as the standard surgical procedure for patients with UC, refractory to medical therapy [1, 2].

The formation of an ileal reservoir or pouch during IPAA, often results in inflammatory changes similar to that of UC [3, 4]. These changes give rise to pouchitis, which is now considered as the most common long term inflammatory complication [1, 3, 5-7]. According to statistics, approximately 30-45% of patients with UC must undergo surgical treatment at some point for this condition [8]. Studies carried out in China estimate a prevalence of UC of up to 11.6 cases per 100,000, as well as over a three-fold increase in the number of cases between the 1980s and 1990s [9]. Likewise, other studies show that the incidence of a first episode of pouchitis increases with time, and that 50% of the patients with recurring episodes develop refractory pouchitis [7].

With increased knowledge and better understanding about the pathophysiological mechanisms involved in the inflammatory process of UC and pouchitis, a series of biological agents have been developed over the past decade [5, 10, 11]. These agents include an antisense oligonucleotide called alicaforsen, which has been designed to inhibit the expression of adhesion molecules involved in the inflammatory cascade of UC and pouchitis [11, 12].

## UC and Pouchitis

UC is categorized as an inflammatory bowel disease (IBD). It is a complex, chronic disease that is characterized by recurring, relapsing and remitting acute ulcerating inflammation of the colorectal mucosa. UC is known for its diffuse, continuous nature and its tendency to be restricted to the colon and rectum. Patients with UC, who fail medical therapy or develop complications such as dysplasia, toxic megacolon, bowel perforation and cancer, eventually require total TPC with IPAA. This surgical procedure involves the removal of the colon and rectum and the construction of a small bowel reservoir (pouch) that is subsequently anastomosed to the anus to reestablish the gastrointestinal tract [1, 8, 13-15].

Although TPC-IPAA is associated with improved quality of life of patients, inflammation of the constructed pouch (pouchitis) is not uncommon. In fact, pouchitis is considered as the most common long term inflammatory complication associated with this surgical procedure [1, 3, 5-8]. According to Shen et al, pouchitis is defined as a nonspecific inflammatory condition in the ileal pouch reservoir [1]. Its development is believed to be associated with contributing factors such as bacterial invasion and faecal stasis [5]. Pouchitis is diagnosed using a combination of clinical symptoms, endoscopic features of acute inflammation and histological evidence of a prominent polymorphonuclear cell exudate [3]. Conventional medical treatment of pouchitis includes antibiotics, steroids and aminosalicylates. This therapy, however, has proven less satisfactory for patients with chronic, recurrent and unremitting pouchitis. Patients with refractory pouchitis eventually undergo further surgery to remove the diseased pouch and resort to the use of a permanent ileostomy [7].

## Intercellular Adhesion Molecule-1 (ICAM-1)

Human ICAM-1 forms part of the immunoglobulin super-family [12, 16]. It is essentially an inducible transmembrane glycoprotein that is expressed on the membranes of the colonic and vascular endothelium, as well as on the cell surface of a subset of leucocytes [12, 16-18]. ICAM-1 expression is upregulated in response to proinflammatory mediators, and this glycoprotein functions by binding to primary counter-ligands on the surface of leucocytes, such as β_2_ integrins, Mac-1 and leucocyte function-associated antigen 1 [12, 16]. Studies have demonstrated increased ICAM-1 expression within the inflamed gut, as well as an increase in circulating concentrations in the blood [16].

## Pathophysiology of the Inflammatory Process in UC and Pouchitis

According to Lightfoot et al, ICAM-1 is responsible for the migration of leucocytes from the areas of inflammation, and it constitutes an active component in the pathophysiology of inflammatory bowel disease [17].

**Figure 1:**
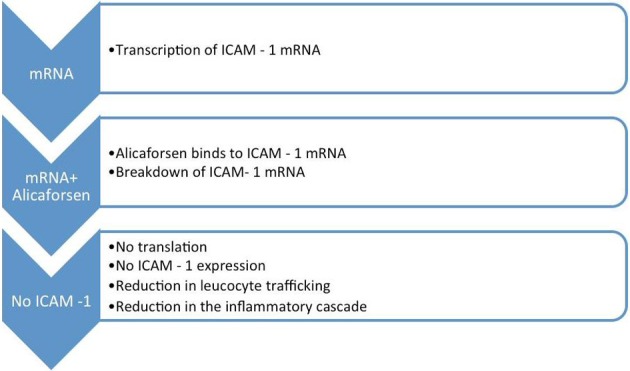
Cascade of events in the inflammatory process.

Leucocyte trafficking in the intestines occurs as a result of the expression of ICAM-1. In order to reach the area of inflammation, leucocytes must be guided by a pathway of adhesion molecules. ICAM-1, along with other similar adhesion molecules, interacts with the counter-ligands on the surface of leucocytes, thereby contributing to the migration of these cells [10]. The first step involves their interaction with the endothelium of the post capillary vessels. In order to slow down the leucocytes that are normally travelling at relatively high speed, the adhesion system is activated, causing them to tether and roll along the endothelium. These processes are subsequently followed by firm adhesion and later migration of the leucocytes from the blood into the inflamed tissue [10, 13, 14, 16] (Fig. 1).

The expression of ICAM-1 is upregulated in response to proinflammatory mediators. This process also occurs along the epithelium of the colon, which further aggravates the local inflammatory process [16] (Fig.1).

## Alicaforsen

The advances in biotechnology, as well as in the research of the pathophysiological mechanisms involved in the inflammatory process of inflammatory bowel disease, have led to the discovery and investigation of a group of biological agents that are capable of inhibiting the expression of molecules that give rise to the inflammatory cascade, especially in UC and pouchitis [3, 10-12].

Alicaforsen constitutes one of the most promising biological agents for the treatment of both UC and refractory pouchitis [7, 10, 12, 16, 18]. It is a 20-base antisense oligonucleotide (5'-GCCCAAGCTGGCATCCGTCA-3') developed by ISIS pharmaceuticals Inc. (Carlsbad, CA, USA), and assigned the code name ISIS 2302 [11-13, 18]. Alicaforsen is considered as a highly selective ICAM-1 inhibitor that down-regulates ICAM-1 mRNA, the messenger RNA responsible for the development and expression of ICAM-1 on the cell surface. ICAM-1 plays a very important role in the inflammatory process associated with UC and pouchitis [7, 11-13, 16].

## Mechanism of Action of Alicaforsen

Alicaforsen is known as an antisense oligonucleotide, because its base sequence is complimentary to the ICAM-1 messenger RNA [19]. Following the transcription of the ICAM-1 mRNA, alicaforsen binds with the same through a process called hybridization. This process renders the ICAM-1 mRNA ineffective and consequently results in its destruction by cleavage [7, 10, 12, 16].

**Figure 2:**
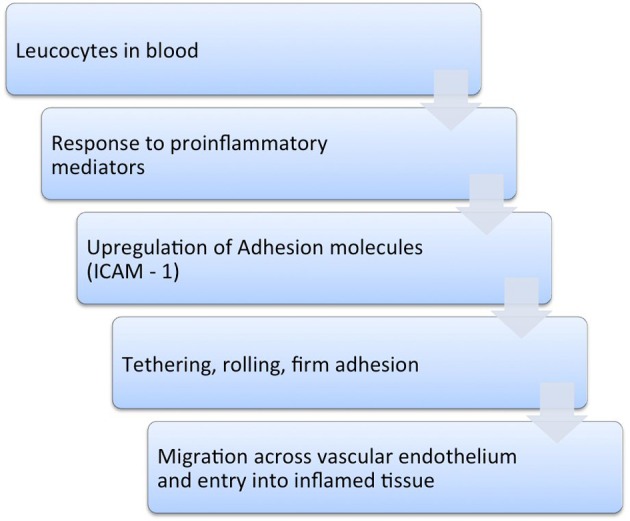
Mechanism of action of alicaforsen.

As a result, translation of ICAM-1 mRNA and subsequent ICAM-1 expression are inhibited. This entire chain of events reduces the migration and trafficking of leucocytes, with a substantial reduction in the inflammatory cascade associated with UC and refractory pouchitis [7, 10, 14, 18] (Fig. 2).

## Studies Asserting the Effectiveness of Alicaforsen

Over the past recent years, clinical studies have been undertaken to assert the effectiveness of the use of alicaforsen in patients with UC and refractory pouchitis. Overall, the results are promising, although more clinical trials in various centers are still required [7, 10, 12, 16, 20, 21] (Table 1).

**Table 1:** Studies Analyzing the Use of Alicaforsen in Pouchitis and Ulcerative Colitis

References	Year	Number of patients	Results	
			Pouchitis	Ulcerative colitis
Miner et al [7]	2004	12	58% remission	N/A
Van Deventer et al [16]	2004	40	N/A	70% improvement in disease activity index
Miner et al [21]	2006	105	N/A	24% complete mucosal healing in 240 mg Alicaforsen group
Miner et al [20]	2006	15	N/A	46% improvement in disease activity index, 33% rate of remission
Van Deventer et al [12]	2006	112	N/A	Low relapse rate

Miner et al carried out an open label uncontrolled study of 12 patients with chronic, unremitting pouchitis. The subjects were treated with 240 mg of alicaforsen enema for 6 weeks. Overall, results showed a 58% remission of pouchitis, with no serious side effects of the formulation [7] (Table 1).

Likewise, Miner et al carried out a randomized, double-blind, active controlled trial, with the objective of evaluating the safety and efficacy of two dose formulations of alicaforsen enema, compared with mesalazine enema for the treatment of mild to moderate left-sided UC. A total of 55 subjects received a nightly enema of 120 mg alicaforsen, while another 50 subjects received 240 mg of the same drug. The treatment lasted for 6 weeks, followed by a 24-week monitoring period. Another group of 54 subjects received a 4 g mesalazine therapy, with the same treatment regime and duration. Results showed that the median duration of response to alicaforsen enema treatment was two to three-fold longer in comparison with that of mesalazine. A 24% complete mucosal healing rate was recorded in the 240 mg alicaforsen group, in comparison with a 17% healing rate in the mesalazine group [21] (Table 1).

Miner et al went on to investigate the bioavailability and therapeutic activity of alicaforsen, administered as a rectal enema to subjects with UC, using an open-label study. In this study, 15 subjects were administered 240 mg nightly doses of alicaforsen for 6 weeks. Results obtained showed that the concentrations of the intact oligonucleotide in mucosal colonic tissue biopsy were significantly higher than those observed in plasma. A 46% reduction in main disease activity index, as well as a 33% rate of remission was observed at the end of the period of treatment [20] (Table 1).

Van Deventer et al carried out a randomized, controlled, double-blind, escalating dose study of alicaforsen enema in 40 patients with active UC. Results showed a 70% improvement in the disease activity index of the patients treated with alicaforsen as compared to a 28% response in patients with placebo. In addition, the results improved in a dose dependant manner, with a high level of statistical significance [16] (Table 1).

Similarly, a phase II dose ranging, double-blind, placebo controlled study of alicaforsen enema was carried out by Van Deventer et al in subjects with left-sided UC. A low relapse rate was recorded, with adequate tolerance to study dosing [12] (Table 1).

## Application of Alicaforsen

According to Dike et al, alicaforsen has received fast-track approval from the Food and Drug Administration (FDA), taking into consideration the significant results obtained in the clinical trials, as well as the safety profile demonstrated with its use [22, 23]. Alicaforsen has also received approval from the European Medicines Agency (EMA) for use throughout Europe. This drug is currently licensed to Atlantic Pharmaceuticals Limited [23].

Overall, the most widely employed method for the use of alicaforsen has been via retention enema. This method provides a direct interaction between alicaforsen and the inflamed mucosa [7, 12, 16]. According to Atlantic Healthcare, alicaforsen can be obtained for treatment, following specifications from an authorized healthcare professional. The recommended dosage is 240 mg (60 mL), once daily via enema. A total of 42 sessions constitute a full treatment course [24].

## Conclusions

The management of UC and pouchitis continues to be a very important area of concern. The development of biological agents has paved the way for the use of new therapeutic strategies in the treatment of these disorders. Alicaforsen constitutes one of the most promising biological agents known to down-regulate the expression of ICAM-1, responsible for leucocyte migration and trafficking during the inflammatory response process. Multicenter studies assert the effectiveness of alicaforsen as a promising therapeutic agent for UC and refractory pouchitis. Although alicaforsen has received fast-track approval, more clinical trials in different centers are required to further validate its use.
